# Tuberculosis outbreak investigation and contact management in a care facility for individuals with disabilities

**DOI:** 10.1186/s12879-026-12980-w

**Published:** 2026-02-27

**Authors:** Asuman Okur, Merve Bulut Adaş

**Affiliations:** 1 Faculty of Medicine Department of Public Health, Giresun University, Giresun, Turkey; 2 Republic of Türkiye Ministry of Health, Rize Provincial Health Directorate, Central Community Health Center, Rize, Turkey

**Keywords:** Pulmonary tuberculosis, Contact screening, Preventive treatment, Disability, Care facility

## Abstract

**Background:**

Tuberculosis (TB) remains a major global health concern, particularly in developing countries. Transmission primarily occurs through airborne aerosolized droplet nuclei.

**Aim:**

The aim of this outbreak investigation was to describe the detection, contact screening process, follow-up, and public health response to a tuberculosis outbreak in a long-term care facility for individuals with disabilities.

**Methods:**

The retrospective descriptive outbreak investigation was conducted on 92 participants, including 55 care recipients and 37 staff members. Data were collected from the Public Health Management System’s TB Module and field records. Age, sex, BCG scar status, TST results, comorbidities, and preventive treatment uptake were recorded. All individuals underwent monthly symptom monitoring, and presumptive cases were referred for hospital evaluation.

**Results:**

A total of four active TB cases were identified among care recipients, including one diagnosed posthumously (culture sample was collected ante-mortem). Twenty-one individuals received preventive treatment, with no subsequent TB cases in this group. One individual who did not receive preventive treatment developed TB five months later. The mean age of diagnosed cases was 34.3 years, all had comorbidities.

**Conclusion:**

Early diagnosis, continuous monitoring, and preventive treatment were integral components of outbreak control in this setting. During follow-up, no additional TB cases were detected among contacts who received preventive treatment. These findings may inform outbreak management strategies in similar high-risk congregate settings. Continuous education for care facility staff and primary healthcare providers is essential to ensure the sustainability of TB control.

## Introduction

Tuberculosis (TB), caused by *Mycobacterium tuberculosis*, is one of the oldest known diseases, with high morbidity and mortality rates. Tuberculosis (TB) is one of the top 10 causes of death worldwide and the leading cause of death from a single infectious agent [1]. Approximately one-quarter of the world’s population is infected with *Mycobacterium tuberculosis*; however, most individuals remain asymptomatic and non-infectious [2].

Despite advancements, TB continues to cause significant illness and death globally. The bacillus can enter the human body through various routes, but transmission most commonly occurs via aerosol transmission. In nearly all cases, TB infection results from the inhalation of droplet nuclei (1–5 μm in size) containing viable bacilli, which remain suspended in the air. Infected individuals release these droplets while speaking, coughing, or sneezing [3, 4]. The widespread prevalence of the disease, the high infectivity of source cases, and prolonged or intense exposure to infected individuals significantly increase the risk of transmission.

Tuberculosis (TB) continues to be a significant public health concern in underdeveloped and developing countries. In these areas, factors such as rapid population growth, socioeconomic challenges, conflict, poverty, malnutrition, substance abuse, and HIV infection contribute to the ongoing prevalence of TB. In developed countries, immigration from regions with high rates of TB is a major factor leading to new cases.

In 2014 and 2015, all World Health Organization (WHO) member states and the United Nations adopted the WHO’s End TB Strategy and the UN’s Sustainable Development Goals, committing to the global goal of ending the TB epidemic [5–7].

In Turkey, TB became an epidemic in the first half of the 20th century and was one of the leading causes of death during that period. While the incidence rate was around 30 per 100,000 population in the early 2000s, this rate had decreased to 14.1 per 100,000 by 2018 [4]. The incidence increases with age and is more common in males. In Rize, the case rate between 2018 and 2020 ranged from 15.8 to 15.1 per 100,000, making it one of the provinces with higher-than-average TB rates in Turkey, following regions such as Istanbul, Thrace, South Marmara, and Western Black Sea [4].

Under the National Tuberculosis Control Program, efforts to prevent and control tuberculosis (TB) are implemented in group living settings where at-risk populations reside. Active surveillance, contact investigations, and systematic screenings -such as those conducted in prisons- are utilized to identify new TB cases [6, 8]. TB patients are highly likely to transmit the bacterium to individuals in their households. In Turkey, between 8% and 9% of TB cases are identified through contact screening [4]. High-risk contacts receive preventive treatment, which has proven to significantly reduce the likelihood of developing TB when taken consistently. With prompt diagnosis and effective treatment, TB is a curable disease. In group living facilities, including nursing homes for the elderly and disabled, orphanages, schools, dormitories, and military barracks, mobile screening teams carry out contact investigations whenever a TB case is identified [4, 6, 8].

The occurrence of TB outbreaks in care homes, which were commonly observed in developed countries at the end of the 20th century and early 21st century, remains noteworthy and relevant to current circumstances in Turkey [9–11].The aim of this outbreak investigation was to describe the detection, contact screening process, follow-up, and public health response to a tuberculosis outbreak in a long-term care facility for individuals with disabilities.

## Methods

### Study design

This study was conducted in a single long-term care facility. It was designed as a retrospective descriptive outbreak investigation. The facility provides full-time care for male adults with disabilities related to mental and neurological disorders. The study population comprised individuals who were receiving and providing services at the facility where TB cases were reported in 2023. A total of 92 individuals—55 care recipients and 37 staff members—were included in the study. No sampling method was applied.

### Tuberculosis diagnosis, contact investigation, and treatment

The diagnosis of active tuberculosis (TB) was established in accordance with the Turkish National Tuberculosis Diagnosis and Treatment Guidelines [8]. Active TB disease was defined as microbiologically confirmed or clinically diagnosed pulmonary TB requiring anti-tuberculosis treatment.

Microbiological confirmation was based on culture positivity (Lowenstein–Jensen solid culture), although culture growth was obtained in only one case. For individuals from whom microbiological specimens could not be obtained, the diagnosis was made based on clinical findings (cough lasting ≥ 2 weeks, fever), radiological evaluation (posteroanterior chest X-ray and thoracic computed tomography demonstrating infiltration or cavitation), and epidemiological linkage. Epidemiological linkage was defined as residence in the same care facility during the same period.

Presumptive case: According to the Turkish National Tuberculosis Diagnosis and Treatment Guideline, a presumptive TB case was defined as an individual with TB-compatible symptoms (particularly cough ≥ 2 weeks) and/or radiographic findings suggestive of tuberculosis, pending bacteriological confirmation.

Latent TB infection was defined as a positive tuberculin skin test (TST) result in the absence of clinical or radiological evidence of active disease [8]. Individuals who met the diagnostic criteria for latent tuberculosis infection were selected for preventive treatment.

Contact screening was performed among both staff members and care recipients of the long-term care facility who were considered contacts of tuberculosis (TB) cases.

Screenings and follow-ups were conducted and evaluated by physicians and staff of the tuberculosis control unit, routinely. Following the diagnosis of the first active tuberculosis case, all care recipients and staff underwent a Tuberculin Skin Test (TST) and a posterior-anterior chest X-ray (PA-CXR). Presumptive TB cases identified during the screenings were referred to a hospital for further evaluation.

The first contact investigation, were conducted in accordance with the Turkish National Tuberculosis Diagnosis and Treatment Guidelines. According to risk group assessment, and screening procedures preventive therapy was initiated for individuals aged < 35 years with a tuberculin skin test (TST) induration of ≥ 15 mm.

Data were collected from the Tuberculosis Module of the Public Health Management System and screening records.

### Timeline

#### Outbreak period

March–August 2023 (from the identification of the index case to the detection of the last case).

#### Contact follow-up plan

The contact follow-up period extended from March 2023 to August 2025. In accordance with the Turkish National Tuberculosis Diagnosis and Treatment Guidelines [8], follow-up examinations were scheduled at the 3rd, 6th, 12th, 18th, and 24th months after initial screening and were conducted as recommended. Only follow-up data up to August 2024 were analyzed.

All contacts were monitored through routine symptom assessments during follow-up visits. Individuals receiving preventive treatment were additionally monitored monthly with laboratory tests, including complete blood count (CBC), liver function tests (AST and ALT), and serum creatinine levels.

#### Retrospective study period of interest

March 2023–August 2024.

#### Data collection period

September 2024–December 2024.

### Statistical analysis

The results were presented descriptively. The variables included medical history, age, sex, current medications, presence of a Bacillus Calmette–Guérin (BCG) vaccination scar, tuberculin skin test (TST) results, and receipt of preventive treatment. Descriptive statistics, including frequency, mean, median were used to summarize the data. Given the closed population and outbreak context, cumulative incidence proportions are reported.

## Results

**Fig. 1:**
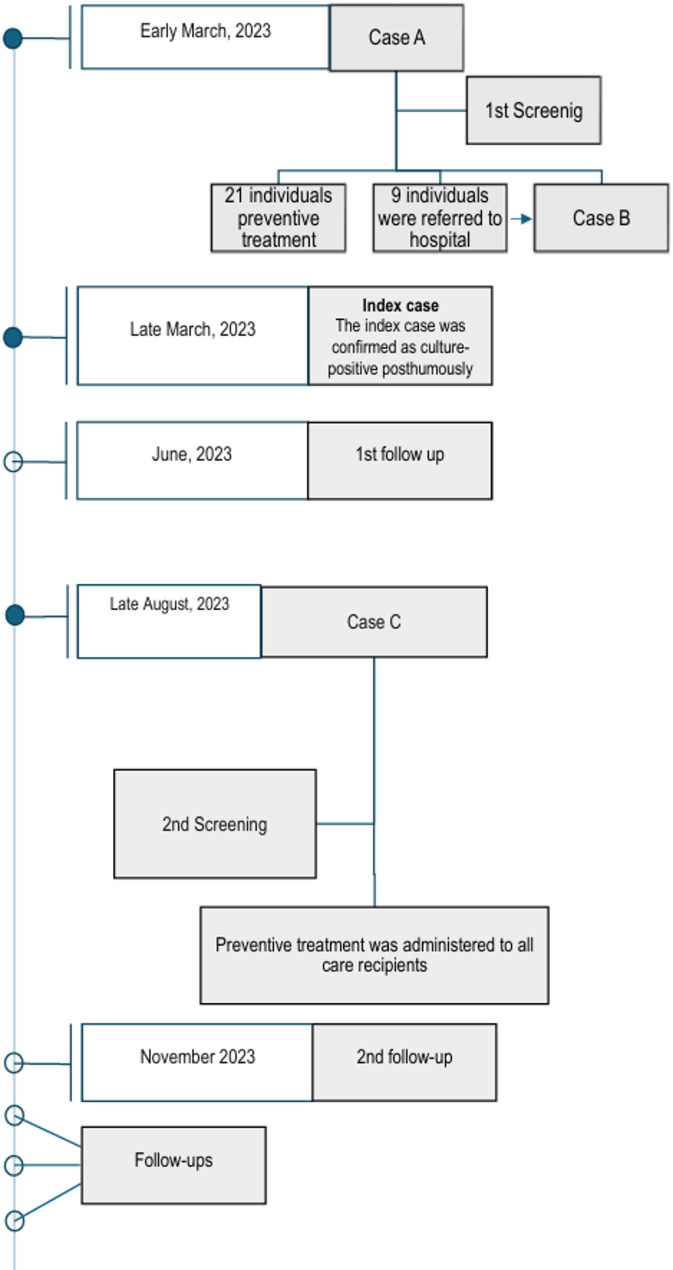
Flow diagram of tuberculosis case investigation and TB outbreak control

When the first active TB case (Case A) was reported, all care recipients and staff at the facility were screened using the tuberculin skin test (TST) and posterior-anterior chest X-ray (PA-CXR). Following the screening, it was found that 60 individuals were within normal limits. A total of 21 individuals were given preventive treatment. This was because their TST result was more than 15 mm or they were considered close contacts under the age of 35. Nine individuals were referred to hospital: one was diagnosed with tuberculosis (Case B) (Fig. 1).

A few days after the screening was completed, the TB culture result of a deceased care recipient who had died before the Case A was identified returned positive. This case was designated the index case. By April 2023, the total number of TB cases identified in the care facility had risen to three, including this result (Fig. 1). All diagnosed patients were treated by the Chest Diseases Clinic.

Follow-up of contacts and individuals receiving preventive treatment continued. In August 2023, a care recipient who had not received preventive therapy was diagnosed with pulmonary tuberculosis (Case C) after experiencing a cough and sputum for approximately 15 days. As this individual was not classified as high risk during initial screening (TST 0 mm) or subsequent evaluations (TST 2 mm) and was over 34 years of age, they had not been administered preventive treatment.

### Key findings

1. The first three cases were diagnosed within a one-month period. One of these was diagnosed posthumously (Index Case) (Fig. 1).
2. As a result of the first screening, 21 individuals were started on preventive treatment (Fig. 1).
3. While follow-ups were ongoing, one individual who had not received preventive treatment was diagnosed with TB in August 2023 (Fig. 1).
4. In the second screening, although no new cases or presumptive cases were identified, preventive treatment was administered to all remaining care recipients contacts. Staff contacts were also offered preventive treatment, and those who accepted received therapy.
5. September 2023- August 2024, no TB cases have developed among those who received preventive treatment.
6. Four individuals were diagnosed with tuberculosis in a care facility where a total of 92 people (care recipients and staff) either lived or worked. The cumulative incidence of active TB in the facility during the period March 2023 to August 2024 was 4.34%.
7. All active TB cases were among care recipients. Among care recipients, the cumulative incidence of active TB during the period March 2023 to August 2024 was 7.27%.
8. The mean age of active TB cases was 34.3 years (median: 33.5).
9. No active TB cases were identified among staff members during the observation period.

## Discussion

This study examined the characteristics of tuberculosis (TB) cases and TB prevention measures in a long-term care facility. It is well established that communal living environments pose a significant risk of developing and transmitting tuberculosis. The fact that all four diagnosed cases occurred among care recipients highlights their increased vulnerability to transmission. This may be due to factors such as prolonged stays in crowded settings, comorbidities and weakened immune systems. The mean age of those diagnosed was 34.3 years. The presence of underlying health conditions, such as epilepsy, cerebral palsy and intellectual disability, that immunocompromising comorbidities may play a role in the development of TB.

Following the diagnosis of Case A in hospital, one additional case (Case B) was identified during the initial screening. Within the same month, three individuals in the same facility were diagnosed with pulmonary TB: one in hospital, one through contact screening and one posthumously via a positive culture result. The individual diagnosed after death was designated the index case. Delayed or post-mortem diagnosis is a common issue in TB outbreaks within care homes. Post-mortem diagnoses reflect delayed detection and gaps in monitoring and follow-up in such settings [12, 13]. In an outbreak investigation by Ijaz et al., retrospective contact tracing identified a 91-year-old woman (Case 3) as the likely source of infection. Despite having shown symptoms for eight months, including a persistent cough, fatigue and progressive pulmonary findings, she was never diagnosed with TB and died without an autopsy [9]. Delayed diagnosis of TB increases both mortality and the risk of community transmission [12]. Although much of the available outbreak literature focuses on geriatric nursing homes, the congregate living structure and prolonged close contact in our facility share similar transmission dynamics. The inability of care recipients to express symptoms or provide sputum samples poses a significant challenge to early diagnosis.

False-negative results from the Tuberculin Skin Test (TST) can occur due to immunosenescence, malnutrition, or comorbid illnesses. In such cases, the Interferon-Gamma Release Assay (IGRA)—a more sensitive and specific test than TST—should be considered as an alternative in selected situations [12, 14]. Beyond awareness, accessibility and feasibility of IGRA are also critical. In the province where this facility is located, IGRA testing is currently unavailable.

Case C was not identified as high-risk during the initial screening or in subsequent assessments and was over the age of 34; thus, preventive treatment was not administered. This case underscores the importance of evaluating the use of more sensitive diagnostic tests in screenings. Narain et al. reported a similar case: a 23-year-old male with intellectual disability and diabetes presented with cough and weight loss but had three consecutive negative TST results. His TB diagnosis was delayed by at least two months [10].

In the same study, regular TST screening and administration of isoniazid (INH) for preventive therapy among infected individuals were found effective in preventing TB transmission and development of active disease in care home settings. Due to 85–90% of elderly patients in such institutions being TST-negative, their susceptibility to infection—and the consequent potential for outbreak—was found to be high [8]. In our study, follow-up was conducted not only through TST but also via chest radiography (PA-CXR), which was effective. However, there was no data available regarding the care recipients’ TST results prior to their exposure to TB.

Following the second screening, preventive treatment was extended to all resident contacts as an outbreak-specific precautionary measure.

Despite no active or presumptive cases being identified in the second screening, initiating preventive treatment for all contacts reflected a cautious and preventive outbreak management strategy. All resident and staff contacts continued to be monitored every three months during the first year and every six months thereafter. Follow-up evaluations included symptom screening, physical examination, and PA-CXR. No additional TB cases were detected after implementation of this precautionary strategy; however, causality cannot be established due to the descriptive design. This finding is consistent with previous studies [10].

Upon reviewing the facility’s ventilation system, it was found that air from the infectious patient’s room circulated into common areas shared with other residents, potentially contributing to the transmission risk [9]. Although a formal environmental assessment was not conducted, review of the ventilation system indicated potential air circulation from the index patient’s room to shared common areas.

## Limitations

This study has several limitations. First, the absence of interferon-gamma release assay (IGRA) testing, drug susceptibility testing and the lack of baseline tuberculin skin test (TST) results limited our ability to distinguish between latent tuberculosis infection and newly acquired infection. In addition, the absence of a comparison group restricts the interpretation of the findings in terms of causality. Finally, due to the observational design, potential confounding factors may not have been fully controlled. Therefore, the findings should be interpreted with caution in light of these limitations. Due to the care recipients’ mental impairment, difficulties were encountered in taking their symptoms and collecting sputum samples. No molecular typing was performed to confirm transmission linkage.

Access to the medical records of the index case could not be obtained. Additionally, no detailed evaluation could be performed regarding the relationship between the facility’s ventilation systems, physical environment, and accommodation conditions with the potential transmission of the disease.

## Conclusion and recommendations

This outbreak investigation underscores the importance of timely notification, systematic screening, and structured follow-up in tuberculosis control within closed and high-risk settings such as long-term care facilities. During this outbreak, no additional TB cases were detected among contacts who received preventive treatment throughout the follow-up period.

The decision to administer preventive treatment to all resident contacts represented an outbreak-specific precautionary public health measure. These findings may contribute to informing contact management strategies in similar congregate settings. Furthermore, this investigation emphasizes the value of reviewing prior illnesses, deaths, and hospitalizations during contact tracing in infectious disease outbreaks in communal living environments.

Given the presence of comorbidities, high transmission risk, inability to express symptoms, and difficulty in providing sputum samples, including such vulnerable populations in routine screening programs would be beneficial for preventing morbidity and mortality.

Continuous training and awareness-raising initiatives targeting care facility staff and primary healthcare workers who serve these institutions are considered crucial for sustained TB prevention efforts.

## Data Availability

The data are available from the corresponding author.

## Abbreviations

BCG: Bacillus calmette–guérin
CBC: Complete blood count
HIV: Human immunodeficiency virus
IGRA: Interferon-gamma release assay
INH: Isoniazid
PA-CXR: Posterior-anterior chest X-ray
TB: Tuberculosis
TST: Tuberculin skin test
UN: United Nations
WHO: World Health Organization
